# Pseudarthrosis of the Calcaneus: Advantages of Regenerative Medicine in the Management of a Rare Entity, A Case Report and Review of Literature

**DOI:** 10.2174/1874325001812010141

**Published:** 2018-03-30

**Authors:** Bouaziz Wajdi, Mohamed Ali Rebai, Walid Baya, Nabil Krid, Wassim Zribi, Hassib Keskes

**Affiliations:** 1 Department of Orthopedic Surgery and Traumatology, Habib Bourguiba University Hospital Sfax - Tunisia; 2 Marechal Leclerc Argentan Hospital - Orthopeadics 47 Rue Aristide Briand, Argentan 61200, France

**Keywords:** Calcaneus, Pseudarthrosis, Nonunion, Malunion, Bone marrow concentrate, Regenerative

## Abstract

**Background:**

The follow-up of calcaneal fractures has shown that they are difficult to manage and lead to multiple complications such as malunion and subtalar osteoarthritis. Pseudarthrosis of the calcaneus is an extremely rare complication, which was described in the literature through case reports.

In the existing literature, only seven studies, including thirteen patients have reported the nonunion. However, to the best of our knowledge, no study elucidates the role of new techniques of regenerative medicine such as Bone Marrow Concentrates (BMC) or Platelet Rich Plasma (PRP) in the management.

**Methods:**

We report a case of a patient with a pseudarthrosis after a calcaneal fracture treated with BMC injection in the non-union site, without the need for surgical approach.

**Results:**

Four months after treatment, the patient was ambulant without support and was completely pain-free. Moreover, after one year the radiological follow up by CT scan showed a satisfactory filling of the non union.

**Conclusion:**

In one case, we try to highlight the advantage of our therapeutic alternatives, which are having a good union while avoiding the complications of surgical approaches and without sacrificing the subtalar joint when it is possible.

## 1 INTRODUCTION

Fractures of the calcaneus are difficult to manage and lead to multiple complications such as malunion and subtalar osteoarthritis [1].

The nonunion is only rarely found, and many studies concerning complications after calcaneal fractures do not describe this complication [2-4].

In the available literature, only seven studies, including thirteen patients have reported the nonunion [2, 5, 6].

However, to the best of our knowledge, there is no study that elucidates the role of new techniques of regenerative medicine such as Bone Marrow Concentrates (BMC) or Platelet Rich Plasma (PRP) in the management.

In this paper, we report a case of a patient with a pseudarthrosis after a calcaneal fracture. The current study highlights the advantages of BMC injection, such as having a good union while avoiding the complications of surgical approaches and without sacrificing the subtalar joint.

## 2 CASE REPORTS

A female patient, aged 40, sustained a closed fracture of the right calcaneus caused by a fall from the stairs. The fracture was a joint-depression type according to the Essex-Lopresti classification, and of type IIA fracture according to the Sanders classification.

After 8 days, the fracture was managed, using percutaneous reduction and fixation followed by an active range of motion exercises and 3 months of non-weight bearing. Initial recovery was without complications.

Seven months later the patient returned to the outpatient department complaining of pain at the heel where she was unable to bear weight.

**Fig. (1):**
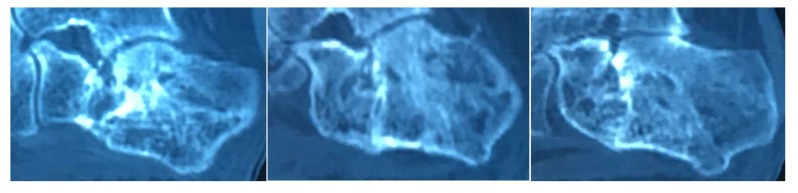
CT-scan showing the nonunion.

Radiological assessment, including CT scan of the foot, revealed a calcaneal non-union with subtalar arthritis (Fig. **1**). The injection of lidocaine in the subtalar joint did not reduce pain.

Under general anesthesia, bone marrow aspirate was sampled from the iliac crest; BMC was prepared by centrifugation and injected at the fractured side.

**Fig. (2):**
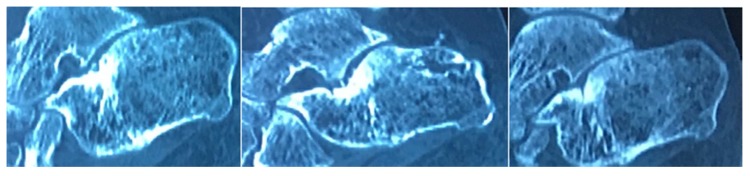
CT-scan 12 months after BMC.

**Fig. (3):**
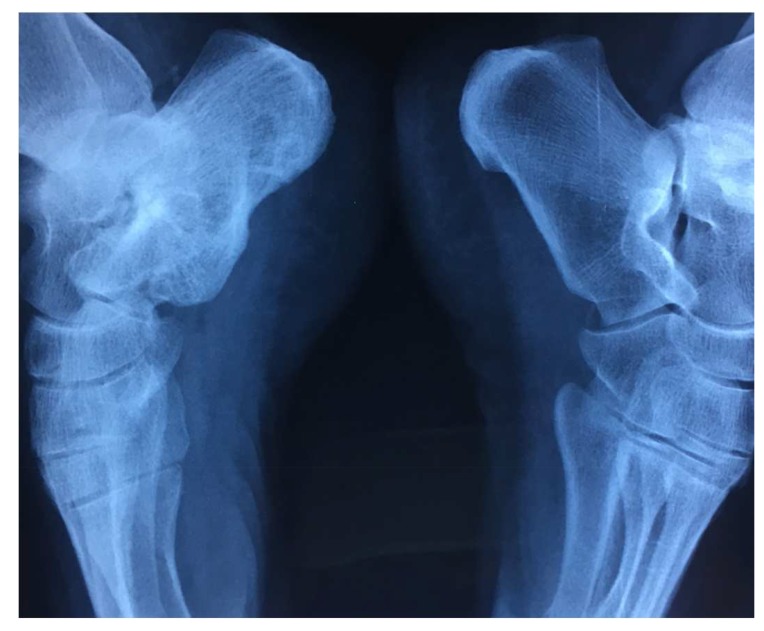
Comparative X ray of both ankle 12 months after BMC.

Four months after the injection, the patient was ambulant without the support and was completely pain-free. Moreover, after one year the radiological follow up by CT scan showed a satisfactory filling of the pseudarthrosis (Figs. **2** and **3**).

## 3 OPERATIVE PROCEDURE

**Fig. (4):**
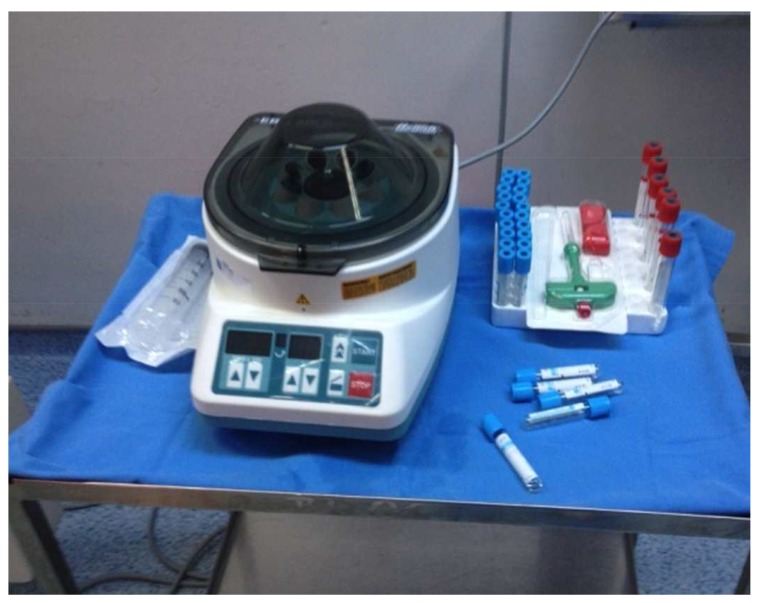
The necessary equipment for BMC.

**Fig. (5):**
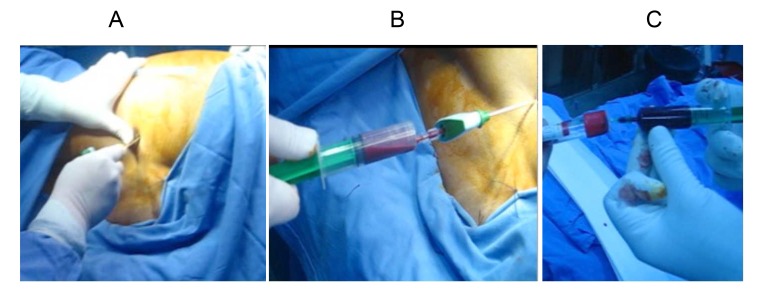
Necessary steps: Insertion of the needle into iliac crest (A).

The operative procedure is simple and a safe method and it does not significantly prolong the time of surgery: under general anesthesia, we harvest the bone marrow from posterior iliac crest using a bone marrow aspirate needle, then the harvested aspirate must be anticoagulated and distributed in glass tubes. The anticoagulant that we use is ACD (acide-citrate-dextrose). The next step is centrifugation (15 minutes at the force of 1500g), and the BMC containing stem cells is then aspirated from the middle layer called “buffy coat”, and injected into nonunion under control of X ray (Figs. **4** and **5A**-**C**).

## 4 DISCUSSION

The nonunion after calcaneal fracture may be underestimated because of rare reporting. The current literature consists of few case reports published in seven studies including thirteen patients (Table **1**). Thomas was the first who reported a case of nonunion after calcaneal fracture in 1993 [5]. Because of underreporting no similarities that could indicate a risk factor of nonunion could be found.

Conservative treatment can be considered as a risk factor of nonunion as shown by Thermann *et al*. who reported an incidence of 10% non-union in their series after conservative treatment [7]. Karakurt *et al*. described smoking as a probable risk factor despite all patients in this report was smokers [3].

According to the reported studies, subtalar arthrodesis, bone grafting, and ORIF have yielded satisfactory results for bone consolidation in the presence of some postoperative complications.

Although strong efforts have been made over the last decade to introduce stem cell and tissue engineering treatment strategies to the field of orthopedics, only few clinical applications are currently available.

Most literature about bone marrow aspirate use in orthopedic trauma has focused on the successful treatment of delayed or non-united fractures of the upper and lower extremities.

However, the characteristics of the nonunion need to be evaluated thoroughly to determine the potential contribution of infection, metabolic abnormalities and mechanical instability, before choosing the appropriate treatment.

In the available literature, the osteogenic ability of BMC has been proofed for long bone, but it was not described for spongy bone defect or non union such as our study.

The results by Jӓger **et al**. [8] in their *in vivo* and *in vitro* study, demonstrated that there is a rationale for a clinical application of BMC in the treatment of osseous defects in long bone.

In his analysis of basic science evidence, Gianakos [9] find that proof-of concept has been established for BMC in the treatment of animal segmental bone defects as both primary treatment strategy and adjunct.

In spite of these promising results, we now have only one obvious fact: the only guarantee of the effectiveness of BMC is its population of osteoprogenitor cells.

Hernigou **et al*.* [10] showed a close relationship between an increased therapeutic effect and an increased number of progenitor cells in bone marrow concentrate.

This number of MSCs in BMC can be affected by harvest and manipulation techniques, and for this reason, we have no standardization in the field of the use of BMC in orthopedics in front of the lack of uniformity in the report of the results, the monitoring of the number of the MSCs and outcomes measurement.

One of the determining factors is harvest location. Hyer and colleagues [11] investigated the MSC yield from different anatomic sites and they found that iliac crest provides the highest number of progenitor cells compared to the calcaneus and distal tibia. Pierini **et al*.* [12] found that the mean number of MSCs from the posterior iliac crest was 60% greater than from the anterior iliac crest.

Hernigou and his team have the widest range of research work aimed at an optimized and highly reproducible BMC technique [13]. In this technique two points must be sited: The marrow should be aspirated in small amounts to reduce the degree of dilution by peripheral blood according to Muschler **et al**. [14], and the fact that the trocar should be turned 45° during successive aspirations to reorient the bevel and aspire from the largest possible space.

Subsequently, Hernigou **et al*.* have demonstrated that syringe size has a significant impact on the number of obtained MSCs and recommended the use of small volume syringes with a large number of aspiration sites when a large number of cells is needed [15].

In the other side, the study by Olivier **et al*.* [16] compared single and multiple site harvesting techniques and they showed that the single-insertion method produced final cellular concentrations and culture results that were not significantly different from those of a multiple-insertion method.

With these studies, the various aspects of the procedure were unveiled, but an evidence-based protocol is still an urgent need.

Our study shows that the new therapeutic alternatives such as BMC may offer the same satisfactory results while avoiding the complications of surgical approaches and without sacrificing the subtalar joint.

However, it is not enough to systematize this treatment because of the small number of patient and the lack of an accurate monitoring of MSCs number.

Controlled randomized studies with a larger sample size and a longer follow-up are required to accurately establish the role of cell therapy in the treatment of the pseudarthrosis of calcaneus.

## CONCLUSION

Pseudarthrosis of the calcaneus is an extremely rare complication.

The current study has demonstrated that BMC injection can lead to a good union while avoiding the complications of surgical approaches and without sacrificing the subtalar joint.

More studies with larger number of patients are needed to systematize this treatment.

## Figures and Tables

**Table 1:** Summary of the literature.

**Study**	**Gender**	**Age**	**Treatment of the nonunion**	**Follow up** **(months)**
Thomas and Wilson [5]	Female	36	Osteotomy, plate, bone graft	3
Thermann **et al*.* [7]	Male	49	Subtalar arthrodesis	62
Gehr **et al*.* [17]	Male	38	Osteotomy, screws, bone graft	2
Karakurt **et al**. [3]	Male	42	Bone graft	8
Zwipp and Rammelt [4]	Female Female	61 45	Subtalar arthrodesis, Calcaneocuboid arthrodesis Subtalar arthrodesis	_ _
Schepers and Patka [2]	Female Male Male	53 49 39	Subtalar arthrodesis Subtalar arthrodesis Subtalar arthrodesis	3 14 6
Kumar [6]	Female	29	Subtalar arthrodesis, screws, bone graft	12
Our report	Female	40	BMC	12
